# Magnetic Configurations in Modulated Cylindrical Nanowires

**DOI:** 10.3390/nano11030600

**Published:** 2021-02-28

**Authors:** Cristina Bran, Jose Angel Fernandez-Roldan, Rafael P. del Real, Agustina Asenjo, Oksana Chubykalo-Fesenko, Manuel Vazquez

**Affiliations:** 1 Instituto de Ciencia de Materiales de Madrid, CSIC, 28049 Madrid, Spain; fernandezroljose@uniovi.es (J.A.F.-R.); rafael.perez@icmm.csic.es (R.P.d.R.); aasenjo@icmm.csic.es (A.A.); oksana@icmm.csic.es (O.C.-F.); mvazquez@icmm.csic.es (M.V.); 2 Department of Physics, University of Oviedo, 33007 Oviedo, Spain

**Keywords:** cylindrical magnetic nanowires, magnetocrystalline anisotropy, magnetochiral configurations, micromagnetic modeling

## Abstract

Cylindrical magnetic nanowires show great potential for 3D applications such as magnetic recording, shift registers, and logic gates, as well as in sensing architectures or biomedicine. Their cylindrical geometry leads to interesting properties of the local domain structure, leading to multifunctional responses to magnetic fields and electric currents, mechanical stresses, or thermal gradients. This review article is summarizing the work carried out in our group on the fabrication and magnetic characterization of cylindrical magnetic nanowires with modulated geometry and anisotropy. The nanowires are prepared by electrochemical methods allowing the fabrication of magnetic nanowires with precise control over geometry, morphology, and composition. Different routes to control the magnetization configuration and its dynamics through the geometry and magnetocrystalline anisotropy are presented. The diameter modulations change the typical single domain state present in cubic nanowires, providing the possibility to confine or pin circular domains or domain walls in each segment. The control and stabilization of domains and domain walls in cylindrical wires have been achieved in multisegmented structures by alternating magnetic segments of different magnetic properties (producing alternative anisotropy) or with non-magnetic layers. The results point out the relevance of the geometry and magnetocrystalline anisotropy to promote the occurrence of stable magnetochiral structures and provide further information for the design of cylindrical nanowires for multiple applications.

## 1. Introduction

The increasing interest in nanomaterials with curved geometry lies in the novel magnetic phenomena observed in those magnetic systems [1]. This can lead to multiple applications which are already being developed currently or are quite promising in the near future. They include magnetochiral phenomena (a consequence of curvature) and other novel effects that open new perspectives not only from fundamental aspects but also in advanced technologies [2,3]. The scientific and technological exploration of three-dimensional magnetic nanostructures is an emerging research field that opens the path to exciting novel physical phenomena, originating from the increased complexity in spin textures, topology, and frustration in three dimensions [4]. 

A particular case is that of nanowires with a circular cross-section. Among other possibilities, the electrochemical route to fabricate cylindrical nanowires inside ordered porous templates is a less-expensive method that offers wide versatility. Ordered arrays of cylindrical nanowires have attracted much interest due to their broad range of applications that go from 3D magnetic information and logic devices, to advanced sensors based on magnetotransport and magnetomechanical responses, spin-caloritronics, microwave, and magnonics, or as novel permanent magnets [5,6]. Advances in smart electronics, robotics, and virtual reality demand electronic skins with both tactile and touchless perceptions for the manipulation of real and virtual objects; here magnetic microelectromechanical systems based on cylindrical nanowires can be used to transduce both tactile and touchless sensing via magnetic fields [7,8]. More recently individual nanowires have been proposed, after being properly functionalized, for biomedical applications in drug delivery and oncological applications, or for nanorobots and swimming nano & microdevices conducted by applied magnetic fields [9,10].

However, most applications are based on the magnetic behavior of individual cylindrical nanowires. The magnetic response of these nanowires can be firstly tailored through their composition (e.g., based on Fe, Co, and Ni as single magnetic elements and their alloys) that determines the cubic or hexagonal crystal symmetry and consequently the magnetocrystalline anisotropy. That anisotropy together with the shape anisotropy finally determines the stable magnetic configurations. In fact, magnetic properties, as domain structure and specific remagnetization processes, are in the origin of most relevant technological applications. 

Complex magnetic configurations are either observed experimentally or/and predicted by micromagnetic modeling. Configurations with magnetochiral components are promoted by cylindrical shapes and include vortex domains with complex 3D transitions between them, helical structures, or skyrmion tubes. The remanent stable magnetic domains are determined by the balance between magnetocrystalline and shape anisotropies. Thus, geometry in the relationship between diameter and length in multisegmented nanowires plays an essential role. Similarly, modulations in composition (e.g., either ferromagnetic/ferromagnetic segments with differential magnetic characteristics or ferromagnetic/non-ferromagnetic metal segments, imposing non-magnetic barriers) significantly influence the domain structure. 

Several advanced experimental techniques have been successfully employed to characterize the magnetic state of individual nanowires. That includes magnetic measurements by Magnetic Force Microscopy (MFM) and Magneto-Optical Kerr Effect (MOKE) sensitive to surface magnetism. Other techniques such as X-ray Magnetic Circular Dichroism combined with Photoemission Electron Microscopy (XMCD-PEEM) allow including information of internal configuration. The distribution of magnetic fields has been successfully achieved by advanced electron holography techniques while tomography methods are emerging to visualize their 3D structure [11]. On the other hand, micromagnetic simulations are required to complement and fully understand the complex magnetic structures in cylindrical nanowires.

As for the reversal process, owing to the large length to the diameter aspect ratio, the magnetization reversal proceeds directionally along the nanowire when the external magnetic field is applied parallel to its axis. Thus, the reversal proceeds overall from one end to the other via the propagation of domain walls. However, most interestingly, point-like singularities commonly labeled as Bloch points, are typically in the middle of domain walls separating axially magnetized domains [12]. 

The aim of this work is to provide a review of the most relevant results obtained by our group on the fabrication, characterization, and properties of ferromagnetic modulated nanowires with tailored geometry and anisotropy. The review is organized as follows: first, the synthesis and fabrication of high-quality nanowires with tailored geometry and magnetic anisotropy are introduced in Section 2. The experimental and modeling results of modulated nanowires with low and high magnetocrystalline anisotropy are presented in Section 3 and Section 4, respectively. Finally, Section 5 presents the discussion and conclusions.

## 2. Materials and Methods

### 2.1. Alumina Substrates

**Figure 1:**
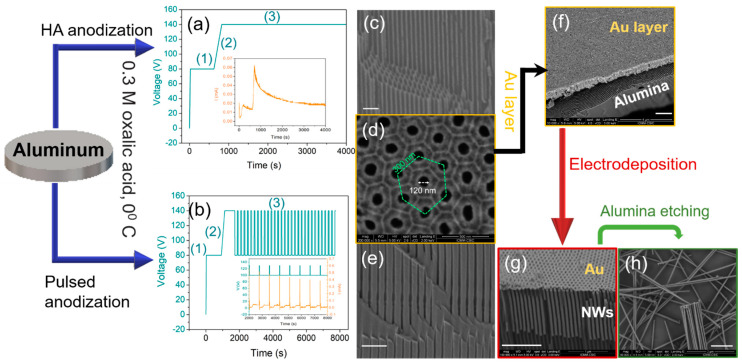
Illustration of the fabrication process. (**a**) Voltage–time transients of hard anodization. The inset shows the current during the anodization, (**b**) Voltage–time transients of pulsed hard anodization, including the first anodization step (1), ramping of voltage (2), and the applied pulses (3). The inset shows a close look of both applied voltage and current transients, during the pulsed anodization, (**c**) Cross-section Scanning Electron Microscopy (SEM) image of alumina templates prepared by hard anodization with pores of uniform diameter of 120 nm, (**d**) Top view SEM image of the bottom side of alumina template presented in (**c**,**e**) Cross-section SEM image of alumina template prepared by pulsed anodization with modulated pores, (**f**) SEM image of an Au layer deposited on the bottom of alumina template, (**g**) cylindrical nanowires deposited by electrodeposition inside the alumina pores, (**h**) individual nanowires released from alumina templates by chemical etching. The scale bar in (**c**,**e**–**h**) is 1 μm.

Anodic aluminum oxide (AAO) templates with pores with diameters between 100–200 nm are prepared by anodization processes from high purity aluminum disks/foils [13,14,15,16,17,18]. The disks are degreased by sonication in acetone (for 10 min) and ethanol baths (for 15 min). Before anodization, the Al discs are electropolished in a mixed solution of HClO_4_:C_2_H_5_OH = 1:3 (*v*/*v*) under a potential of 20 V for 3–5 min. In order to fabricate the templates with pores of uniform diameter over 100 nm, the Al disks are anodized by hard anodization process in oxalic acid 0.3 M solution with 5% of ethanol in volume, at 0–1 °C, following the steps illustrated in Figure 1a. First, a constant voltage of 80 V is applied for 400–600 s (step 1) to produce a protective aluminum oxide layer at the surface of the disc which avoids breaking or burning effects during the second and third steps. In the second step, the voltage is steadily increased (0.07–0.08 V/s) until the highest applied voltage of 120–140 V is reached and kept constant for 3600 s (step 3). The resulting pores with diameters of about 120–130 nm and lengths of about 60 μm (Figure 1c) are distributed in a hexagonal order (Figure 1d) [19,20]. 

The modulated pores are fabricated by pulsed anodization [21,22] using the same acidic electrolyte as for the pores with uniform diameter, where step (3) in Figure 1a is replaced by voltage pulses (Figure 1b). Depending on the anodization parameters (voltage pulses, time) different types of modulations can be obtained. The modulated AAO pores presented in Figure 1e are obtained by applying hard anodization pulses of 100 V and 130 V for 100 s and 5 s, respectively.

An alternative method used in our laboratories for obtaining AAO pores (straight and modulated) with smaller diameters involves the use of a sulfuric acid solution. 

The pulse anodization of aluminum can also be done by replacing the oxide electrolyte with H_2_SO_4_ electrolyte. Periodic pulses, alternating a low and high potential pulse are applied, where the duration of each pulse determines the length of anodized segments at the given applied potential. The modulation of nanopores has been achieved by two-step anodization of aluminum discs in a 0.3 M H_2_SO_4_ at 0 °C temperature. The first anodizing step was performed at a constant potential of 25 V for 16 h [23,24].

**Figure 2:**
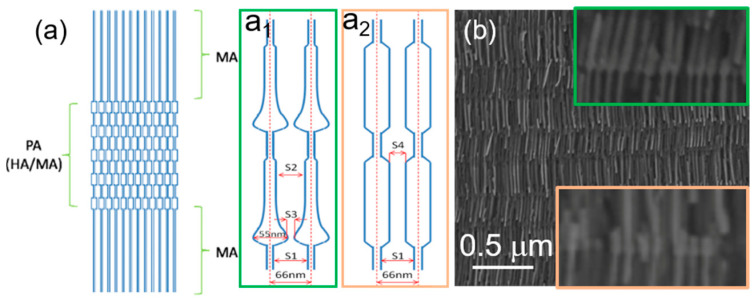
(**a**) Schematic illustration of nanowire geometrical features using modulated diameter within exponential (**a_1_**) and squared (**a_2_**) pulses template (Adapted with permission from ref. [23]. Copyright 2014 IOP Publishing Ltd.), (**b**) cross-section SEM image of FeCo nanowires with modulated pore diameter formed by pulse anodization in H_2_SO_4_ electrolyte. The insets show the slight change in the geometry determined by the shape of the voltage pulse.

After chemical etching of the anodized section, the modulated nanopores are synthesized in the 2nd anodization step by periodically applying pulses of 25 V—mild anodization (MA) pulses, and 35 V—hard anodization pulses (HA) (Figure 2a). The geometry of the nanochannels is controlled not only by the anodization time, voltage, or bath temperature but also by the shape of the HA pulses. The applied voltage pulses with exponential (Figure 2a_1_) and square (Figure 2a_2_) shapes produce slightly different modulation of the nanochannels (Figure 2a,b). The resulting cylindrical modulated pores are formed by segments with diameters of around 22 and 35 nm, while the center-to-center inter-wire distance is kept constant at 65 nm.

### 2.2. Samples Deposition

After the anodization process, the remaining aluminum substrate is chemically etched by a mixed solution of CuCl_2_·2H_2_O and HCl. The alumina barrier layer is removed and the pores are enlarged using a H_3_PO_4_ solution (5 wt. %). Before depositing the nanowires inside the pores, an Au layer is sputtered on the backside of the AAO template (Figure 1f) to serve as a working electrode for electrodeposition. The magnetic nanowires (Figure 1g) can be released from the AAO template by using a mixed solution of CrO_3_ and H_3_PO_4_ (Figure 1h).

The magnetic nanowires are grown mainly by potentiostatic electrodeposition into the pores of AAO templates. The deposition is done at suitable potentials in a three-electrode electrochemical cell equipped with an Ag/AgCl reference electrode, a Pt mesh counter and an Au layer sputtered on the backside of the AAO template, acting as a working electrode. The technique allows controlling the composition of the deposited material, from a single element to alloys or multi-segmented nanowires [25,26,27,28,29]. The electrolytes and parameters used in electrodeposition are presented in Table 1.

Following the procedure presented in Section 2.1, four types of nanopores were obtained. By filling them by electrodeposition, magnetic nanowires mirroring the shape of the pores were produced.

**Figure 3:**
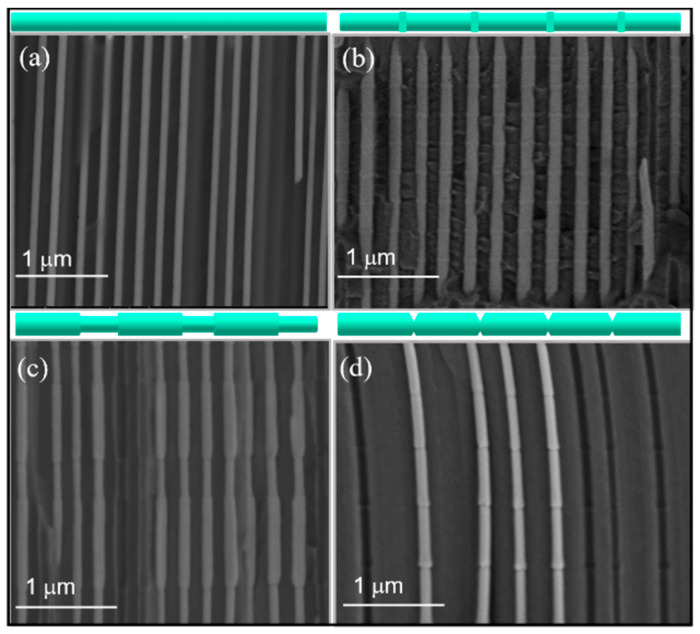
SEM lateral view images of (**a**) uniform (**b**) bamboo-type (**c**) modulated in diameter and (**d**) notched nanowires.

Figure 3 presents SEM cross-section images of (a) FeCo nanowires with a uniform diameter of 120 nm (b) bamboo-type FeCoCu nanowires (c) FeCoCu modulated nanowires with alternating segments of 110 and 130 nm in diameter, and (d) Ni nanowires with notches along their length.

### 2.3. Characterization Methods

Magneto-Optical Kerr Effect (MOKE). The single nanowires have been measured by a Kerr effect magnetometer NanoMOKE TM 2 from Durham Magneto Optics Ltd. (Durham, UK) under a maximum applied field of ±500 Oe. Each hysteresis loop measured by MOKE is the result of 1000 averaged loops. Once the nanowires are dispersed on a silicon substrate, a scan of the surface was carried out by SEM in order to ensure that the MOKE measurements are focused on individual wires and not on several at once.

Magnetic Force Microscopy (MFM). MFM measurements were performed in a Cervantes system from Nanotec Electrónica (Madrid, Spain). The use of amplitude modulation (AM) and the two-pass modes with a phase-locked loop (PLL) enabled tracking the resonance frequency of the oscillating cantilevers. BudgetSensors Multi75M (BudgetSensors, Sofia, Bulgaria) and Nanosensors PPP-MFMR (NANOSENSORS, Neuchatel, Switzerland) probes were used in these experiments. Parallel topographic images were taken to check the diameter modulation periodicity.

X-ray Magnetic Circular Dichroism combined with Photoemission Electron Microscopy (XMCD-PEEM). XMCD-PEEM measurements were performed at the CIRCE beamline of the ALBA Synchrotron Facility (Barcelona, Spain) using an ELMITEC LEEM III (Clausthal-Zellerfeld, Germany) instrument with an energy analyzer [30]. The samples were illuminated with circularly polarized X-rays at a grazing angle of 16° with respect to the surface, at the resonant L3 absorption edges of Fe (708 eV), Co (778 eV), and Ni (851 eV) for the FeCoCu, CoNi, and Ni wires, respectively. The emitted photoelectrons (low energy secondary electron with ca. 1eV kinetic energy) used to form the surface image are proportional to the X-ray absorption coefficient and thus the element-specific magnetic domain configuration is given by the pixel-wise asymmetry of two PEEM images sequentially recorded with left- and right-handed circular polarization [31]. 

**Figure 4:**
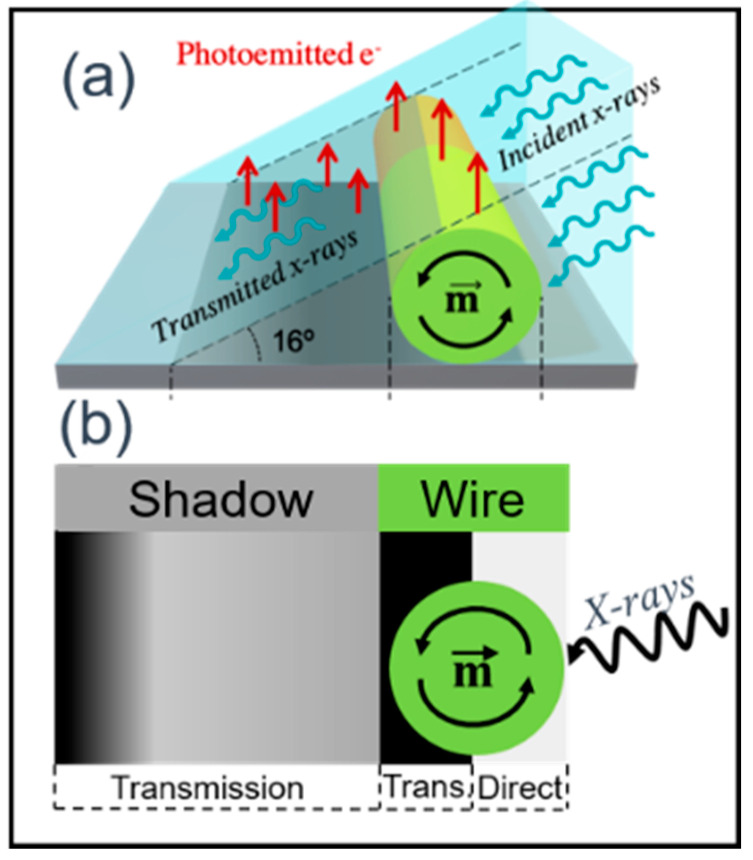
Schematic illustrations of (**a**) the principle of the dual sensitivity of Photoemission Electron Microscopy (PEEM) to detect direct photoemission and transmission data using X-ray Magnetic Circular Dichroism (XMCD) as a contrast mechanism, (**b**) magnetic contrast observed for direct photoemission and transmission. Adapted with permission from ref. [21]. Copyright 2016 Royal Society of Chemistry.

In this arrangement of the setup (Figure 4a), an amount of X-ray photons is transmitted through the nanowire, generating photoemission from the Si substrate. Since the transmitted intensity depends on the relative alignment of the nanowire magnetization and the X-ray helicity, the photoemission from the substrate in the area shadowed by the nanowire does as well. Therefore, by analyzing the circular dichroic or pseudo-magnetic contrast formed in transmission in the shadow area, information about the magnetization configuration in the bulk of the wire can be obtained [21,32]. Notice that dark contrast in the transmission is equivalent to bright indirect photoemission since the absorbed and transmitted X-rays are complementary. XMCD-PEEM thus offers the possibility to obtain both the magnetic structure of the surface and the core of the cylindrical structure (Figure 4b). The projection of the local magnetization on the photon propagation vector is determined, the domains with magnetic moments parallel or antiparallel to the X-ray polarization vector appear bright or dark in the XMCD image while domains with magnetic moments at a different angle have an intermediate grey contrast [20,21,29].

### 2.4. Micromagnetic Simulations

The understanding of 3D magnetic structures and their dynamics requires complementary micromagnetic modeling. In this work micromagnetic modeling of individual nanowires has been carried out using a finite-difference discretization scheme implemented in mumax3 software [33]. The material parameters and the crystal structure are detailed in Table 2. 

## 3. Magnetic Configurations of Cylindrical Nanowires with Large Shape Anisotropy

### 3.1. Magnetic Domain Configuration in Nanowires with Uniform Diameter

The magnetism of cylindrical nanowires is determined mainly by their shape (i.e., geometry) and magnetocrystalline anisotropy. A full understanding of the magnetization reversal process of individual nanowires and their arrays is essential to design and develop novel applications. 

The soft magnetic nanowires, with *fcc* or *bcc* crystal symmetry, exhibit reduced crystalline anisotropy, lower than the axial shape anisotropy [40,41]. The elongated shape of the wire induces a natural axial anisotropy via the magnetostatic energy which favors a high stability of axial magnetic states [42]. Due to the strong magnetic shape anisotropy, these nanostructures present high coercivity and remanence when magnetized along their long axis. 

**Figure 5:**
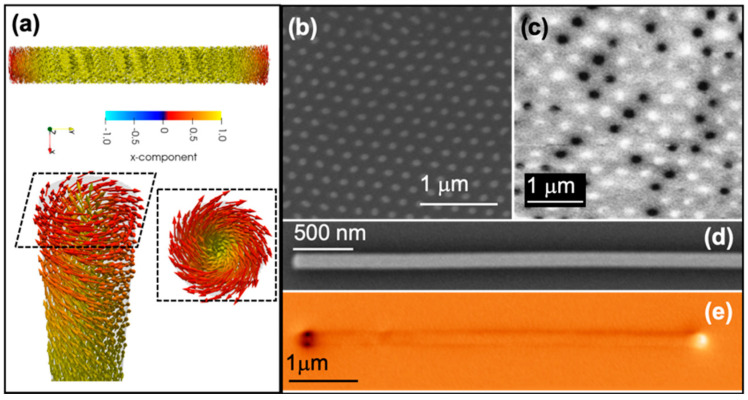
(**a**) Micromagnetic simulation of an individual FeCo nanowire presenting a longitudinal magnetization with vortices at the ends (top panel). Close-up images of the vortex structures are presented in the bottom panel, (**b**) SEM top view image of alumina template filled by magnetic nanowires, (**c**) Magnetic Force Microscopy (MFM) image of Ni nanowires embedded into the template (Reprinted with permission from ref. [49]. Copyright 2007 American Physical Society), (**d**) SEM image of a single CoNi nanowire, (**e**) MFM image of Co_35_Ni_65_ nanowire.

The magnetic state of nanowires with cubic structures, i.e., Ni, Fe, and Py, is characterized by a predominant magnetization component along the nanowire axis with two open vortices at the nanowire ends (Figure 5a) which minimize the magnetostatic energy. The magnetization reversal takes place by domain wall propagation. The type of domain walls by which the nanowire demagnetizes is determined by the nanowire geometry (diameter) and material. While the thin nanowires demagnetize by transverse domain wall, the large-diameter nanowires demagnetize via Bloch point (previously called vortex) domain wall [29,42,43,44].

The incorporation of new elements into the system modifies both the structure and magnetic response of nanowires. One of the relevant materials is a Co-based alloy which presents different magnetic configurations as a function of the crystallographic structure, highly influenced by the preparation parameters (electrolyte, temperature, deposition, pH) [45]. In the case of NiCo wires, the magnetic properties are tuned through the composition of the alloy [38,46,47]. For less than 50% Co, the shape anisotropy predominates, allowing for a variation of coercivity as a function of composition, while for the content of Co > 50% the magnetocrystalline anisotropy becomes predominant and the magnetic properties are mainly determined by the crystallographic phases. A promising nanowire alloy is FeCo due to its high saturation magnetization and elevated Curie temperature, magnitudes that make it relevant in most technological applications, and specifically for a novel family of permanent magnets [36,48]. 

The first experimental investigations on shape anisotropy-dominated nanowires were done by MFM on Ni nanowires with *fcc* structure, 180 nm in diameter, embedded into alumina templates. The measurements were done at the top of the alumina template filled with magnetic nanowires. To remove the roughness at the top of the alumina substrate which can influence the MFM measurements the samples are mechanically polished. An example of a mechanically polished alumina template with the ends of the nanowires reaching the surface is presented in Figure 5b.

The MFM image in Figure 5c was taken after the sample and the tip were saturated in a negative magnetic field (black contrast). The white/black contrast corresponds to nanowires with the magnetization oriented up or down, respectively. When the magnetization of the nanowires points parallel/antiparallel to the tip field direction, we obtain black and white contrast, respectively. A simple magnetic phenomenological model allowed determining the magnetostatic interactions which strongly influence the remanence of the array [49].

The single-domain state in an individual nanowire (Figure 5d) with low magnetocrystalline anisotropy is presented in Figure 5e. The MFM image presents an individual Ni_65_Co_35_ nanowire with 120 nm in diameter where a uniform contrast is observed along the nanowire length, indicating a single longitudinal domain state. The bright/dark contrast at the ends of the nanowire is due to the presence of magnetostatic charges.

### 3.2. Magnetic Configurations in Nanowires with Tailored Geometry

In order to be used in 3D nanotechnological applications, e.g., data storage, sensing, magnetomechanical actuation, or bio applications, the control of domain wall dynamics, nucleation, mobility, and pinning is paramount [1,50,51]. To pin the domain walls at certain positions along the nanowire length, several strategies have been considered. The approach consists of the creation of potential wells and barriers where the domain wall gets pinned. To do so, the nanowire geometry is altered creating constrictions along the length, artificial notches, anti-notches, defects, or diameter modulations that act as pinning sites for the domain walls. 

Here, we discuss two types of geometrical constrictions: anti-notches (bamboo-type nanowires) (Figure 3b) and modulations in diameter (alternating diameters) (Figure 2b and Figure 3c). The considered modulated nanowires are made of FeCo alloys with high saturation magnetization (~1.8–2 T). 

The first investigations were done by MFM on modulated Fe_30_Co_65_Cu_5_ nanowires with small diameters deposited in modulated pores prepared by pulsed anodization in sulfuric acid (Figure 2a_2_,b). 

**Figure 6:**
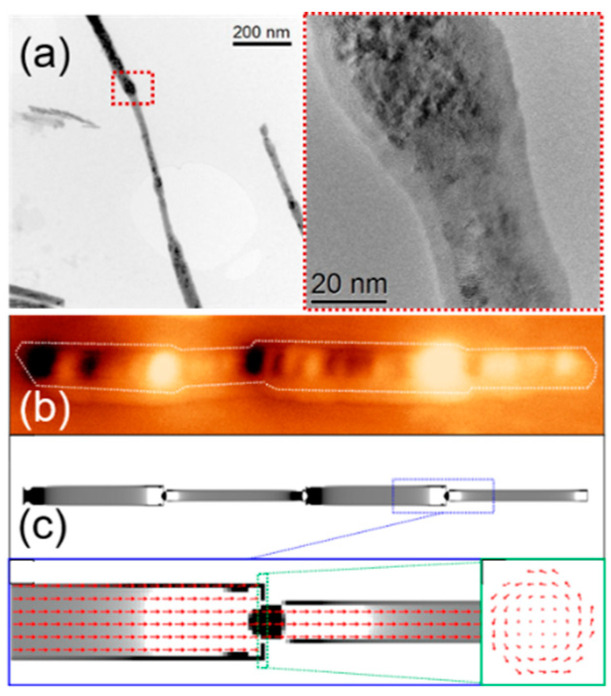
(**a**)-(left), HRTEM images of a modulated nanowire, (right), a close-up image of the area highlighted with a red square in (**a**)-(left). (**b**) MFM image of an isolated FeCoCu modulated nanowire (**c**) Simulated configuration at remanence for a modulated wire with two thick and two thin parts, equivalent to the experimental case. Adapted with permission from ref. [24]. Copyright 2015 IOP Publishing Ltd.

Figure 6 presents the morphology, the magnetic configuration, and micromagnetic simulations of an isolated FeCoCu modulated nanowire. The High-Resolution Transmission Electron Microscopy (HRTEM) images in (a) present the morphology of an isolated wire formed by alternating segments of few hundreds of nanometers with two distinct diameters 22 and 35 nm. The HRTEM data also revealed the crystallographic structure of the two types of segments. The nanostructures present a cubic (*bcc*) crystalline structure in both segments, but with higher texture along the (110) direction in the segments with a larger diameter.

The magnetic configuration determined by MFM (Figure 6b) presents a single domain state that gives rise to strong contrast at the ends of the wire as well as at the transition region between segments of different diameters. The experimental data are supported by micromagnetic simulations (Figure 6c) where apart from the overall longitudinal configuration of magnetization, a curling effect is obtained at the transition regions between segments of different diameters. Moreover, the weaker bright/dark contrast, observed along segments, is correlated with higher roughness observed in modulated nanowires with smaller diameters [24].

A more detailed and defined geometry has been obtained for the nanowires with diameters over 100 nm (Figure 3) fabricated in the modulated pores obtained by pulsed anodization in oxalic acid (Figure 1b). The magnetization reversal of individual FeCoCu nanowires with diameters over 100 nm and the influence of tailored periodical geometrical modulations have been studied by the Magneto-Optical Kerr Effect (MOKE) [19]. 

**Figure 7:**
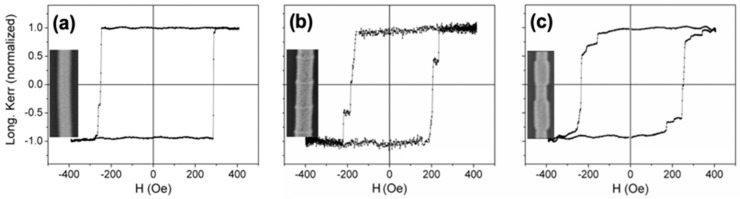
Magneto-Optical Kerr Effect (MOKE) hysteresis loops for uniform diameter (**a**) bamboo-type (**b**) and modulated diameter (**c**) nanowires. The insets show the SEM images of individual nanowires. Adapted with permission from ref. [19]. Copyright 2015 IOP Publishing Ltd.

Figure 7 shows the hysteresis loops for homogeneous diameter (a) and modulated in geometry (b and c) nanowires measured with the laser spot focused in the center of the nanowire. For the nanowire with a uniform diameter (a), a square hysteresis loop is observed with a sharp transition between two stable magnetic states at remanence through a single giant Barkhausen jump, suggesting the existence of a single domain structure with axial magnetization [38,52]. For the bamboo-type nanowires in (b), two abrupt and symmetric magnetization jumps are observed in each branch of the hysteresis loop. In the case of the modulated nanowire (c), the hysteresis loop shows the existence of a main Barkhausen jump together with several additional ones of smaller amplitude. The presence of several magnetization jumps in the modulated nanowires suggests the existence of metastable magnetic states during the magnetization reversal that could be correlated to their particular diameter modulation [19,52]. The MOKE measurements done at different spots along the nanowire’s length and their angular dependence indicate that the demagnetization process takes place by the propagation of a single vortex domain wall which eventually is pinned at given modulations with a slightly higher energy barrier [19].

A clear picture of the magnetic configurations in these two types of modulated in geometry nanowires was obtained by employing advanced microscopy techniques like Magnetic Force Microscopy (MFM), X-ray Magnetic Circular Dichroism combined with Photoemission Electron Microscopy (XMCD-PEEM), and Electron Holography. XMCD-PEEM offers the possibility in the cylindrical geometry of nanowires to obtain a full mapping of magnetic configuration. Due to the partial transmission of the X-ray beam through the wire, the magnetic state of the nanowire core is mapped onto the substrate, providing simultaneous information of the magnetization distribution at the surface (direct photoemission from the wire) and inside the nanowires (photoemission from the substrate) (Figure 4a).

In addition, electron holography (EH) supplies information on the magnetic flux distribution of the internal magnetic structure and the stray fields outside the nanowires. The technique provides a quantitative analysis of both the crystallographic structure and the magnetic properties obtained on the same area, and at the nanoscale [22,53,54].

**Figure 8:**
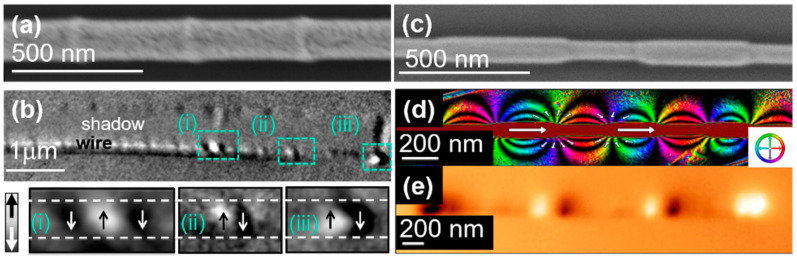
(**a**) SEM image of a bamboo FeCoCu nanowire, (**b**) XMCD-PEEM image of bamboo-type FeCoCu nanowire (**a**) oriented perpendicular to the incident X-rays (Adapted with permission from ref. [21]. Copyright 2016 Royal Society of Chemistry). The insets show a closer view at the local magnetic configuration marked in (**b**) by green dashed squares, (**c**) SEM image of a modulated FeCoCu nanowire, (**d**) magnetic flux images of modulated FeCoCu nanowire (**c**) reconstructed from the magnetic phase shift images, (**e**) MFM image of a modulated FeCoCu nanowire. Adapted with permission from ref. [22]. Copyright 2016, American Chemical Society.

Figure 8a,b present the morphology and XMCD-PEEM measurements of a bamboo-type FeCoCu nanowire with anti-notches placed at about 400 nm along the nanowire axis. The X-ray diffraction data revealed that the FeCoCu wires present a *bcc* polycrystalline structure. The XMCD image in Figure 8b is characterized by a modulated profile along the entire length. The intensity profile in the surface region contains bright/dark local contrasts matching the position of each modulation along the length. Between those local regions, the surface shows a reduced grey contrast, characteristic of a longitudinal magnetization orientation perpendicular to the X-ray propagation vector. The enhanced magnetic contrast can be observed at the end of the wire and at given modulations, as shown in the insets of Figure 8 (i), (ii), and (iii) suggesting vortex-like domain walls pinned at the anti-notches. In the shadow, we get information about magnetization orientation inside the nanowire. A uniform grey contrast is observed in the main region of the shadow along the whole length of the nanowire. The vanishing grey contrast of the main region of the shadow reveals the longitudinal orientation of the magnetization inside the nanowire [21,55].

A quantitative magnetic characterization has been performed on individual diameter-modulated FeCoCu nanowires with alternating segments of 100 and 140 nm (Figure 8c) by electron holography and MFM [22]. The analysis shows that the diameter-modulated geometry of the wires induces the formation of vortex-like structures and magnetostatic charges at the border between segments with different diameters, modifying the axial alignment of the magnetization in large-diameter segments. Furthermore, the magnetostatic charges influence the stray field distribution, inducing a flux-closure stray field configuration around large-diameter segments and keeping the demagnetizing field parallel to the nanowire’s magnetization around small diameter segments (Figure 8d). The holography data complements the MFM data unveiling the origin of bright and dark contrast observed along the nanowire (Figure 8e). 

A similar study was performed on two sets of Ni tri-segmented modulated nanowires, with two different diameters of D_1_ = 160 nm (145 nm) and D_2_ = 130 nm (100 nm) and lengths of L_1_ = 9.7 μm (6 μm) and L_2_ = 1.6 μm (5.5 μm) [56]. The experimental and modeling data revealed that the vortex domain wall nucleates and propagates along the nanowires. In the case of the nanowire formed by a thin segment encapsulated between two larger segments, the domain wall is trapped at the junction between the large and narrow diameter. 

**Figure 9:**
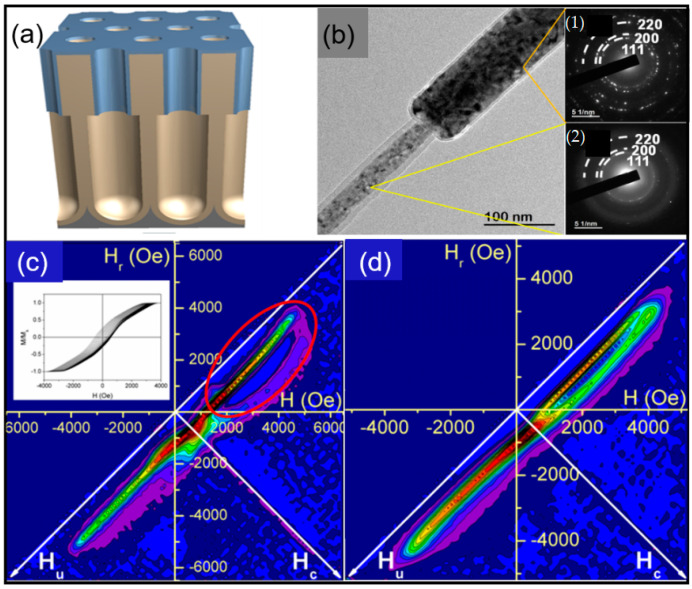
(**a**) Schematic view of highly ordered alumina template with modulated pores, (**b**) HRTEM image of a bi-segmented Ni nanowire. The insets show the Selected Area Electron Diffraction (SAED) patterns corresponding to the wide and narrow segments of the nanowire shown in (**b**,**c**) First-Order Reversal Curve (FORC) distribution diagram for the bi-segmented Ni nanowire arrays. The inset shows the hysteresis curves used for calculating the respective FORC diagram (**d**). FORC distribution diagram for the Ni nanowire arrays with 80 nm in diameter (Adapted with permission from ref. [57]. Copyright 2019 Springer Nature.).

By employing a combination of Atomic Layer Deposition (ALD) and anodization, Prida et al. [57,58] synthesized alumina templates with hexagonally ordered pores having one well-defined geometrical modulation in the diameter (Figure 9a). Ni and Fe_50_Co_50_ nanowires were grown into the tailored alumina membranes by electrodeposition to replicate the geometry of the alumina templates with bi-segmented pores (Figure 9b). The nanowires with two distinct diameters (30 and 80 nm) show a polycrystalline crystallographic cubic structure, *fcc* (220) for Ni nanowires and *bcc* (110) for FeCo nanowires, respectively (Figure 9b-inset).

A uniaxial magnetization easy axis was determined for the bi-segmented nanowires with the magnetization reversing through the propagation, in steps, of a domain wall due to the geometrical modulation. This can be seen in the hysteresis loops and in the First-Order Reversal Curve (FORC) data (Figure 9c,d) of Ni nanowire arrays. The FORC diagram obtained for bi-segmented Ni nanowires (Figure 9c) shows a FORC distribution shape associated with a magnetic nanowire array with predominant shape anisotropy. The diagram is characterized by a single branch, which spreads widely parallel to the interaction field axis. However, due to the sharp modulation, this branch splits in two at different values of coercive field (see the red markings in Figure 9c). A FORC diagram for nanowires with a homogeneous diameter (80 nm) was also obtained (Figure 9d) showing only one branch parallel to the interaction field axis. The magnetic experiments show the two-step magnetization reversal process, ascribed to the segments of different diameters, in both Ni and FeCo nanowire arrays [57,59].

### 3.3. Micromagnetic Simulations of Nanowires with Tailored Geometry

**Figure 10:**
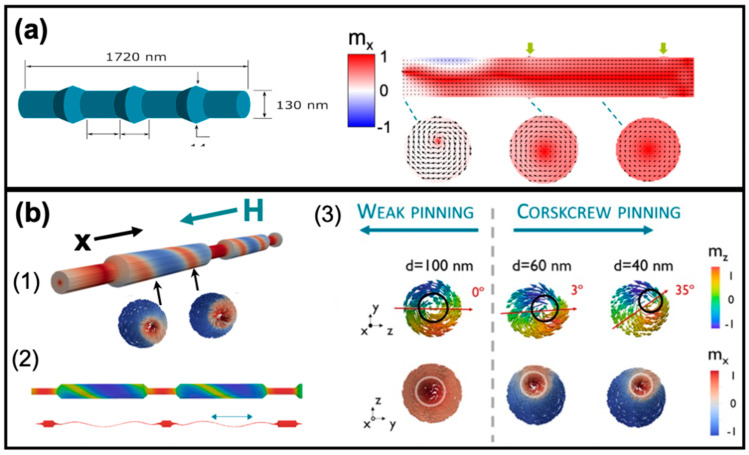
(**a**) Geometry of a FeCo nanowire with anti-notches (bamboo-type nanowire) on the left side and, a snapshot of one end of the nanowire at the remanent state on the right (Adapted with permission from ref. [21]. Copyright 2016 Royal Society of Chemistry.). The green arrows indicate the position of the anti-notches. The dashed lines show the location of the cross-sections. (**b**) (1) The magnetization at the surface of a modulated nanowire with segments of 130 nm in diameter and a smaller diameter d in a magnetic state before magnetization switching. The cross-section taken at the positions marked by arrows shows the skyrmion tube state. (2) The core of the skyrmion describes a helicoidal curve inside the segments of larger diameter. The segments of minor diameter remain uniformly magnetized. (3) magnetization configurations in the cross-sections of the segments with larger diameters in nanowires with segments of smaller diameters. Adapted with permission from ref. [39]. Copyright 2018 Royal Society of Chemistry.

Micromagnetic simulations are the necessary tool to assist the design of the geometry and material properties of nanowires with the aim to induce the pinning/unpinning processes of domain walls and to understand the mechanisms behind them. In this section, we discuss several possibilities of geometrical constrictions in FeCo cylindrical nanowires based on anti-notches (Figure 10a) and modulations in diameter (alternating segments with different diameters) in Figure 10b. The micromagnetic parameters are listed in Table 2.

The geometry and the remanent state of an individual FeCo nanowire with anti-notches (bamboo-type) are shown in Figure 10a. Overall, the magnetization adopts an axial orientation (except for the regions close to the anti-notches) determined mainly by the shape anisotropy of the nanowire which is orders of magnitude larger than the magnetocrystalline anisotropy of FeCo. In addition, this magnetic configuration shows open vortices at each end of the nanowire. Along the nanowire surface, the magnetization shows a smooth curling that reduces the formation of magnetostatic charges at the positions of each anti-notch (marked by green arrows). This deviation of the magnetization from its axial orientation agrees with the periodic contrast reported by MFM [55] and XMCD [21] data for FeCo-based bamboo nanowires.

Figure 10b presents the magnetization configuration, prior switching, at the surface of a single modulated polycrystalline FeCo nanowire with alternating segments of larger diameter, 130 nm, and minor diameter (d = 40–100 nm) [39]. The demagnetization process in this nanowire begins in the segments of the larger diameter by means of the nucleation of the open vortex structures with arbitrary vorticity at the constrictions. These structures unpin from the constriction and propagate first inside the wide diameter segments and later inside the small diameter segment. When the difference between diameters is small the propagation in both segments, although consequent and not simultaneous, takes place at the same field value (“weak pinning”).

For a larger difference in diameters between the two segments, i.e., strong pinning of magnetic vortex structures nucleated at constrictions, the first stage of the demagnetization process consists only in the propagation of the structures inside the segments of larger diameter. At a second stage (at more negative fields), the vortex structures de-pin from the constrictions and propagate inside segments of small diameter, i.e., the magnetization switches its axial orientation.

Importantly, the open vortex structures at the constrictions between large and small segments are formed with arbitrary chirality. As the magnetic field is increased further, the magnetic moments in the outer nanowire shell rotate towards field direction forming a spiral skyrmion tube (see Figure 10b-(1)). This also can be seen from the magnetization configurations in large segment cross-sections in Figure 10b-(1), the magnetization structure is formed by a magnetic skyrmion with the core pointing against the direction of the magnetic field and the shell pointing parallel to it. Once the demagnetization is completed in the large-diameter segments, the skyrmion structure remains pinned at the constrictions. Importantly, the skyrmion center along the segment is not located in the center of the nanowires but describes a spiral (see Figure 10b-(2)), a corkscrew magnetization pattern [39]. The formation of spiral for vortex/skyrmion tube center is a consequence of the minimization of magnetostatic charges (magnetic poles) at the constriction. These charges are typically created at the constrictions and are redistributed along the nanowire length to minimize the energy. Arrot et al. [60] argue that these charges are proper to all magnetic nanowires but they should be particularly visible in nanowires with large saturation magnetization where the minimization of magnetostatic energy is favorable. Similar topologically protected structures and the core-screw surface structure have been recently reported in FeNi nanowires with chemical barriers and microwires [61,62,63]. 

### 3.4. Magnetic Configuration of Individual Nanowires with Chemical Notches

**Figure 11:**
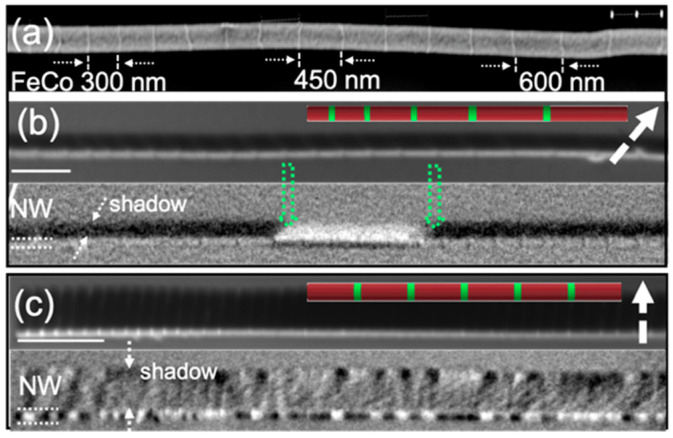
(**a**) SEM image of a multisegmented FeCo/Cu nanowire. Sequences of X-ray Absorption Spectroscopy (XAS) (above, with an indication of the beam direction: dashed white arrows) and PEEM images of (**b**) multisegmented FeCo/Cu nanowire with a variable length of FeCo segments, and (**c**) multisegmented FeCo/Cu nanowire with constant length of FeCo segments. The scale bar is 1 μm.

A controlled magnetization reversal can be achieved apart from geometrical constrictions, by chemical notches placed along the nanowire’s length (Figure 11a). 

The engineering of the magnetic properties can be done not only through the architecture of the nanostructure (combination of materials-layers) but also by controlling their crystallography or composition and interfaces. Their fabrication is usually achieved by two methods: a sequential deposition where two electrolytic baths are involved or by using a single electrolytic bath and varying the electrodeposition potential or current density to obtain a different composition in each layer [64]. The first method allows full control over the composition of individual layers/segments, while the second one produces better interfaces between additional layers.

FeCo(x)/Cu (30 nm) nanowires were grown by electrodeposition in a three-electrode cell, at room temperature, from a single electrolytic bath (electrolyte (1) in Table 1). The pH value of the electrolyte was maintained at about 3.0. The applied potential, versus the Ag/AgCl reference electrode, was alternately pulsed between −0.6V to deposit Cu and −1.8 V for different time periods to deposit the FeCo layers [65,66]. To be measured individually, the nanowires were released by chemical etching from the template. 

Figure 11 displays the magnetic configuration, measured by XMCD-PEEM of two individual FeCo/Cu multisegmented nanowires. Above each stack of XMCD-PEEM images, we show the direct X-ray Absorption Spectroscopy (XAS) image at the Co L_3_ absorption edge for chemical identification of the Cu segments. The XMCD-PEEM images present contrast both in the wire (dotted lines labeled NW) and in the shadow, due to the photoemission from the substrate after transmission through the wire core. Figure 11a,b shows the SEM image (a) and chemical (top panel) and magnetic contrast (bottom panel) (b) of a FeCo/Cu multisegmented nanowire with 120 nm in diameter, fixed Cu layers of 30 nm, and increasing length (from left to right) of FeCo segments (~200–1000 nm). The XMCD contrast along the wire suggests three longitudinal magnetic domains with magnetic moments pointing along the nanowire axis and separated by two domain walls pinned at the Cu constrictions as evidenced by green dotted arrows. Furthermore, the homogeneous contrast of opposite orientations between the nanowire and shadow observed in all the individual segments indicates a uniform axial magnetization at the nanowire surface and core.

A different magnetic configuration is presented in Figure 11c where, although the nanowire composition is similar to that presented in (a), the magnetic landscape is different. The images presented in Figure 11c present the chemical and magnetic contrast of a FeCo/Cu nanowire with constant FeCo and Cu segments of 250 nm and 50 nm, respectively, and 165 nm in diameter. 

The top panel in Figure 11c shows the XAS image at the Co L_3_ absorption edge for chemical identification of the different segments (modulated contrast on the wire and in the shadow). The magnetic contrast profile (Figure 11c-bottom panel) presenting bright/dark on the wire or dark/bright in the shadow matches the position of magnetic layers, suggesting the presence of a single vortex structure in each FeCo layer. The measurements indicate that the magnetic moments are pointing parallel (white contrast on the wire/dark in the shadow) or antiparallel (dark/white contrast in the shadow) to the polarization vector.

The examples presented above offer a comprehensive picture of the role played by the architecture of the nanostructure: on one hand, we have strongly coupled FeCo segments across the thin Cu layers which orient the magnetization along the long axis of the nanowire (Figure 11b) while in the second example, the reduced magnetostatic coupling due to the lower shape anisotropy determined by the short lengths of the segments and larger Cu spacer, makes each segment behaving as a single nanodot with the magnetization in a single vortex state (Figure 11c).

### 3.5. Manipulation of Magnetization Reversal by Magnetic and Electric Fields

The full control over the domains and domain walls in nanowires with circular cross-sections requires the determination of the conditions for their efficient and minimal manipulation by magnetic fields and spin-polarized currents.

**Figure 12:**
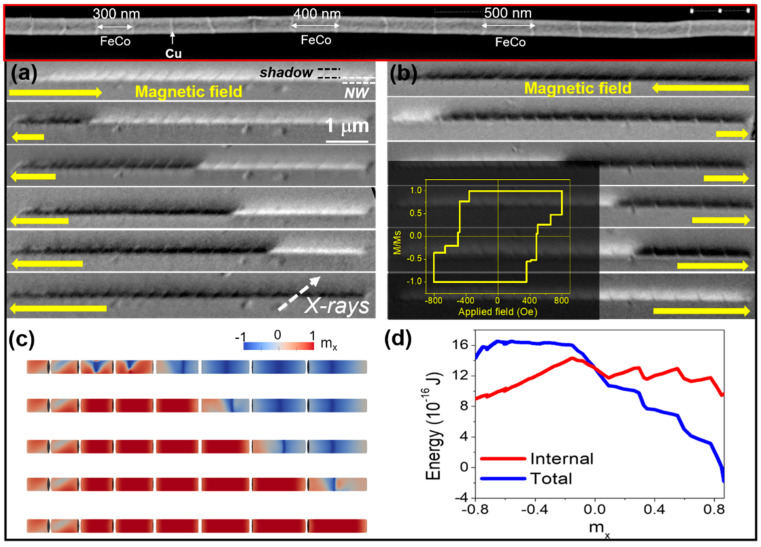
(**a**,**b**) Selected PEEM images under increasing applied field along the leftward (**a**) and rightward (**b**) polarity. The inset in (**b**) shows the reconstructed hysteresis loop. (**c**) Simulated magnetization configurations showing the sequential reversal during the reversal process. (**d**) Total and internal magnetic energies in FeCo/Cu nanowire as a function of the longitudinal magnetization component evaluated by the micromagnetic simulations during the ascending branch of the hysteresis loop. Adapted with permission from ref. [65]. Copyright 2018 American Chemical Society.

The magnetization reversal following a ratchet effect was observed in multisegmented nanowires with increasing segment lengths presented in Figure 12 [65]. The unidirectional propagation, irrespective of the longitudinal field direction, is experimentally observed and confirmed by micromagnetic simulations in Fe_35_Co_65_/Cu multisegmented nanowires with fixed Cu layers of about 30 nm and variable lengths of FeCo segments (200–1000 nm). The magnetization reversal has been observed to proceed in few irreversible jumps at which magnetization reverses (as observed by MFM sensitive to surface mostly and detected by MOKE) as well confirmed in the whole nanowire segments as seen in XMCD-PEEM measurements (Figure 12a,b). The reversal process is always unidirectional, irrespective of the external field direction, initiating at the end of segments with a shorter length. Such ratchet effect originates in the broken symmetry induced by the shape anisotropy of increasing length of the FeCo segments and, like in a domino effect, it is promoted by the magnetostatic coupling between adjacent segments.

The micromagnetic simulations (Figure 12c,d) reveal a complex process where, although the switching is sequential from one segment to another, the magnetization process inside each segment takes place by the formation of vortices and skyrmion tubes followed by the final collapse of the internal core. The formation of skyrmion tubes with opposite chirality and strong topological protection may constitute the origin of pinned magnetic states. The plotted energy in Figure 12d displays the ratchet-like potential created by the increasing shape anisotropy, exchange energy, and pinning sites [65].

Reports on domain wall motion and control by means of electric current and thermo-magnetic switching in cylindrical nanowires are scarce and their analysis is limited [12,67].

**Figure 13:**
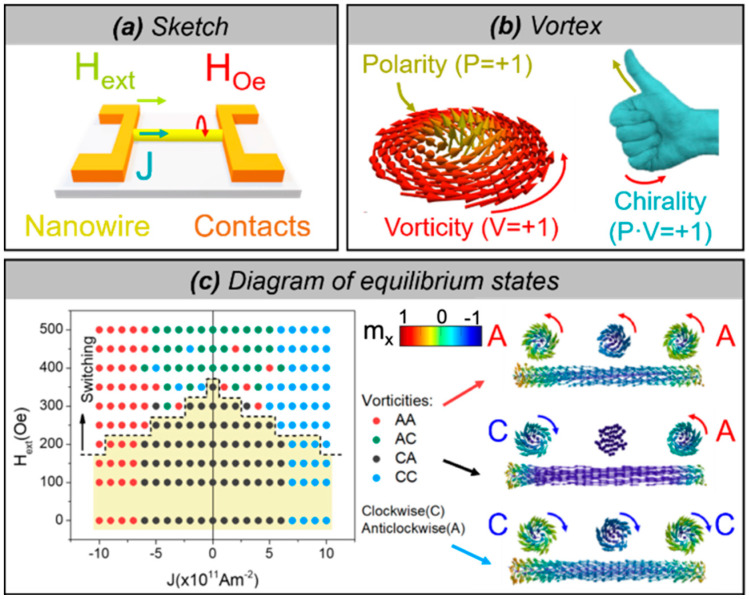
(**a**) Schematic illustration of a contacted nanowire in an experiment on magnetization dynamics. A spin-polarized current J flowing through the nanowire generates an Oersted field H_oe_ which adds to the external field H_ext_. (**b**) The polarity and vorticity of a vortex are respectively defined by the directions of the magnetization in the inner core, and the sense of the rotation around this core (anticlockwise (A) or clockwise (C)). Their product determines the chirality which is equivalently determined by right/left-hand rule as right/left-handed vortex or anticlockwise/clockwise vortex. (**c**) Left side—a diagram of vortex stationary states as a function of the applied field and current. C/A stands for clockwise/anticlockwise vorticity. The threshold for the switching field of the axial component is indicated by the dashed line. Below this line (yellow-shaded region) no magnetization switching occurs, i.e., the shaded and non-shaded regions correspond to vortices with polarity +1 and −1 respectively. On the right side, three representative configurations of the magnetization for H_ex_ = 0 and vortex states AA, CA, and CC. Adapted with permission from ref. [68]. Copyright 2020 American Physical Society.

Here, we introduce the results of our micromagnetic modeling on the manipulation and control of the polarity and vorticity of magnetic vortex structures (originating from their precursors at the nanowire ends at remanence) in a Fe_20_Ni_80_ cylindrical nanowire with a diameter of 100 nm by the simultaneous application of external fields and spin-polarized currents (Figure 13a). The remanent state is a uniform axial magnetic domain with open curled structures (Figure 5a, precursors of vortex domain wall or magnetic domains) with opposite chirality at each end of the nanowire (Figure 13c with J = 0). In all cases, vortices consist of an axially magnetized core (its direction defines the polarity of the vortex) and a curling around the core (whose sense of rotation determines the vortex vorticity) as in the example in Figure 13b. The product of integer numbers (polarity by vorticity) is known as chirality. See [68] for modeling and further details.

The diagram of stationary states is presented in Figure 13c and demonstrates the possibility for manipulation and control of the resulting vortex structures (i.e., both magnetization at the core and shell) with fields and currents. The axial magnetization component is only switched (at a critical value of the field) with the assistance of both the external field and current. For magnetic fields below the switching field, only the vorticities can be controlled by the current density and magnetic field. For the currents and fields for which the switching of the magnetization at the inner core occurs (above the dashed line), the sense of rotation (vorticity) of both vortex structures is regularly reversed from anticlockwise/clockwise (AC) to CA and vice versa for low currents. Since the Oersted field is not large enough to set the rotation sense in this case, they are determined by the direction of the magnetization and the resulting torque, and therefore the reversed pattern is found. In addition, there are some low currents for which CC and AA are found when the magnetization is switched. This ambiguity is not observed for higher current values, for which the chirality is fully determined by the Oersted field, either CC or AA. Conveniently, setting the current and field to zero values does not alter the vortex pattern and hence it is completely controlled. Notice that below the dashed line the chirality is determined by the vorticity, whereas above the dashed line the polarity is reversed, and the chirality is therefore determined by both the polarity and the vorticity. The switching of both polarity and vorticity, preserves the chirality of the initial remanent state, whereas the switching of only one of them leads to the chirality switching. Furthermore, the whole process takes place in a few nanoseconds, consequently, excessive Joule heating could be prevented by the use of short field and current pulses of this duration.

## 4. Magnetic Configurations of Cylindrical Nanowires with Large Magnetocrystalline Anisotropy

While in the case of nanowires with a cubic crystalline structure the shape anisotropy and magnetostatic interactions mostly determine the overall magnetic response [34,69], in the case of Co-based nanowires with a hexagonal crystallographic structure the magnetocrystalline anisotropy plays an important role as its easy axis can orient at different angles with respect to the nanowire axis. In Co nanowires, the crystalline structure can be tuned by varying the growth parameters such as applied voltage, pH, dimensions (diameter, length), or annealing and deposition under external magnetic fields. It has been shown that pH-controlled electroplating enables the switching between *fcc-* and *hcp*-Co phases, which modifies the magnetization easy axis from parallel to perpendicular to the wires [45,70,71]. Furthermore, the magnetic properties of Co nanowires can also be tuned by adding different materials to the Co system. One of these materials, CoNi, allows the design of the crystalline structure and magnetic configuration in the nanowire through the composition of the alloy [20,38]. 

**Figure 14:**
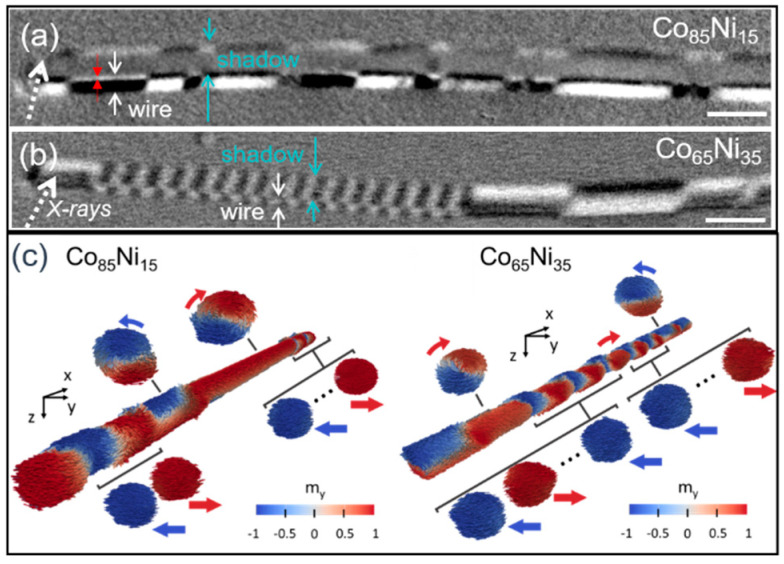
XMCD-PEEM images of (**a**) Co_85_Ni_15_ and (**b**) Co_65_Ni_35_ nanowires. The scale bar is 1 μm, and the arrows (dash white) indicate the incident X-ray beam, (**c**) micromagnetic simulations of Co_85_Ni_15_ (left) and Co_65_Ni_35_ (right) nanowires. Adapted with permission from ref. [20]. Copyright 2017 American Physical Society.

Figure 14a shows the XMCD image of a Co_85_Ni_15_ nanowire taken at a nearly perpendicular configuration of the X-ray propagation vector with respect to the nanowire axis. The marked regions in the images labeled “wire” and “shadow” correspond respectively to photoemission from the nanowire surface and that from the substrate after transmission through the wire volume. The surface (wire, marked by white arrows in (a)) consists of a sequence of segments with an azimuthal magnetic configuration of opposite contrast, i.e., vortex-like structures with alternating chirality. The contrast observed in Figure 14a is interpreted by the schematic illustrations in Figure 4. Due to the cylindrical geometry of the nanowire and the grazing angle of the X-ray with respect to the sample surface (Figure 4a) only a certain region of the nanowire surface (the bright contrast region) is directly exposed to the X-rays while the rest is only sensitive to the transmitted beam (the dark contrast region) [21]. This contrast, arising from the transmitted beam at the wire’s surface can be seen in Figure 14a, marked by red arrows. The second type of contrast is found in the shadow area due to the transmitted X-rays through the volume of the nanowire. The uniform grey contrast in the middle of the shadow (corresponding to the core of the wire) is an indication of the magnetization pointing perpendicular to the beam, i.e., along the nanowire axis. Notice that for the same magnetic moment orientation, opposite contrast is expected between direct and transmitted signals [20,21].

A more complex magnetic configuration is found in Co_65_Ni_35_ nanowires (Figure 14b). The XMCD-PEEM image taken at Co L_3_-edge shows two types of magnetic structures. On one hand, on the right side of the wire, a sequence of segments with opposite contrast similar to those in Figure 14a is observed. The increased contrast in the shadow indicates that the circular/vortex structures with ~1 μm in length, observed at the surface of the wire, penetrate into the volume, reducing the width/diameter of the longitudinal core. On the other hand, on the left side of the wire, a sequence of segments with shorter periodicity and alternating contrast (bright/dark at the surface, dark/bright in the core) is observed. The contrast in the shadow is opposite to that at the surface and remains constant in the transversal direction, showing that the magnetization state is homogenous along the circular cross-section of the wire. This is interpreted to correspond to alternating periodic transverse domains with the component of magnetization in the perpendicular direction to the nanowire axis. The width of the observed transverse domains is estimated to be about 150 nm and is very regular [20]. 

The coexistence of the metastable hybrid states in CoNi wires is confirmed by micromagnetic simulations which, furthermore, offers a better understanding of the magnetization processes. The simulations of Co_85_Ni_15_ nanowire are shown in Figure 14c (left side) where the equilibrium state is formed by a series of vortex and transverse domains along the nanowire. The magnetic landscape shows mainly vortex domains with opposite chirality and different lengths separated by shorter transverse domains with opposite directions close to the ends of the nanowire. For the Co_65_Ni_35_ nanowire (Figure 14c (right side)) a more complex domain structure is obtained. Again, a hybrid magnetic structure of transverse and vortex domains is observed along the nanowire. However, the transverse domains occupy a larger fractional volume, while the vortex domains mostly appear close to the ends. 

### Magnetic Nanowires with Modulated Anisotropy

The control and stabilization of domains and domain walls have been observed in multi-segmented nanowires [61,62,72,73] by alternating magnetic segments of different magnetic behavior or with non-magnetic metallic layers. These nanostructures can provide active channels for domain wall pinning or spin-wave manipulation. The multilayers formed by non-magnetic/magnetic layers can be used for domain wall pinning or confinement of specific domains [65]. The nanostructures formed by ferromagnetic/ferromagnetic segments offer the possibility to produce a system with alternating magnetocrystalline anisotropy which can pin domain walls between the segments or nucleate and stabilize them due to the influence of the neighboring material [72,73,74].

In this context, Co-based alloy nanowires are good candidates for tailoring designed magnetic anisotropies and consequently, magnetic behavior. The low magnetocrystalline anisotropy of cubic materials combined with the high anisotropy of the hexagonal symmetry of Co allows tailoring the magnetic properties of the system. 

Multi-segmented CoNi/Ni nanowires with tailored alternating magnetic anisotropy have been fabricated and investigated with the aim to control the occurrence of different states by using the effect of confinement and interaction between segments. While in the case of Ni segments, due to the lack of magnetocrystalline anisotropy a single axial domain state is expected due to the predominant shape anisotropy, in the case of CoNi, due to the strong magnetocrystalline anisotropy energy constant of Co, the magnetic configurations can be tuned with respect to the composition (the strength of magnetocrystalline anisotropy and its easy axis) [73]. 

Co_85_Ni_15_/Ni wires with 140 nm in diameter, 1000 nm long Ni segments, and CoNi segments between 600 and 1400 nm in length were synthesized via electrochemical route. HRTEM data reveal that the Ni presents an *fcc* structure while the Co_85_Ni_15_ segments show an *hcp* crystalline structure oriented along the [010] direction. The magnetic configuration was imaged by XMCD-PEEM in the demagnetized state and at remanence after magnetizing the wires axially and perpendicularly. 

**Figure 15:**
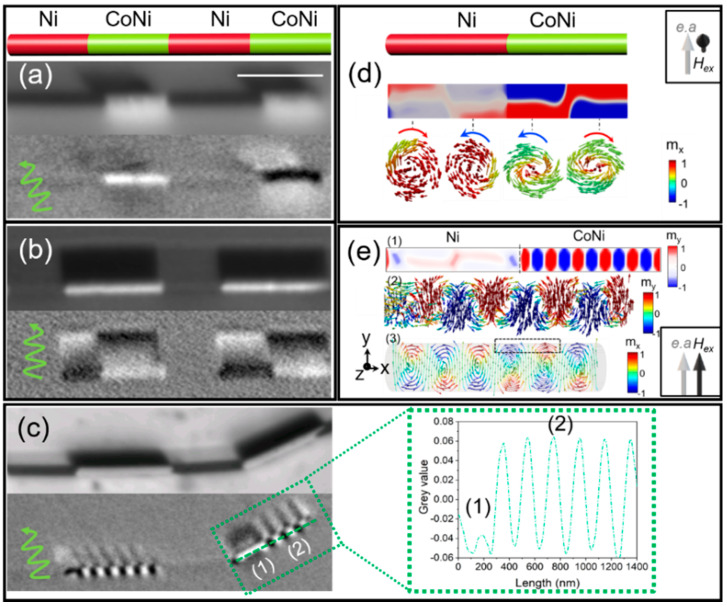
Chemical contrast (upper images) and XMCD-PEEM (lower images) contrast at Co L_3_-edge of CoNi(x)/Ni(1000 nm) multisegmented NWs with (**a**) x = 600 nm, (**b**) x = 1200 nm and (**c**) x = 1400 nm. The inset in (**c**): XMCD profile of the CoNi segment at the right side in (**c**) presented at the position marked by the dashed horizontal green line. (**d**) Micromagnetic simulations of multi-segments with the applied magnetic field perpendicular to both, nanowire and magnetocrystalline easy axis (e.a.). (**e**) Micromagnetic simulations of multi-segments with the applied magnetic field parallel to the magnetocrystalline easy axis and perpendicular to the nanowire. Scale bar: 1 μm. Adapted with permission from ref. [73]. Copyright 2020 American Chemical Society.

Figure 15 presents the XMCD-PEEM images taken at the Co-edge, of CoNi/Ni multisegmented NWs with lengths of the CoNi segments of 600 nm (a), 1200 nm (b), and 1400 nm (c) after the previous demagnetization in a perpendicular magnetic field. In the shorter CoNi segments (600 nm) the image presents two types of contrasts: bright (dark in the shadow) or dark (bright in the shadow) suggesting single vortex states with different chiralities in different segments. In the CoNi with 1200 nm in length up to three vortices with alternating chirality (contrasts) are formed inside each segment (Figure 15b) while in the NWs with the longest CoNi segments (1400 nm), we observe the occurrence of either vortices or periodic transversal domains (Figure 15c). The inset of Figure 15c shows the XMCD profile over the CoNi segment marked by a dashed green square in (c). At the left side of the CoNi segment, first a vortex domain with a length of about 250 nm is formed (1) followed by the periodic transversal domains (2). The width of the transverse domains is estimated from the XMCD contrast profile across the wire (at the position of the green dashed line) to be about 105 nm (inset Figure 15c) [73]. 

The micromagnetic simulations (Figure 15d,e) reveal the origin of different magnetic configurations in the multisegmented nanowire. Although the Ni segment is a nearly single domain state, its magnetization presents a small magnetization curling at the surface. Its magnetic state is formed prior to the formation of magnetic structures in CoNi and determines the state of it. Interacting Ni segments (separated by short CoNi segments) prefer the same vortex chirality while the almost non-interacting ones (separated by longer CoNi segments) prefer the formation of the alternating chirality. As seen in Figure 15d, the vortex chirality observed in CoNi domains mimics the chirality formed in the Ni segment in order to avoid magnetostatic charges at the transition. Furthermore, the remanent states of CoNi segments depend on the direction of the previously applied field. For the magnetic field applied perpendicular to both the nanowire and the anisotropy easy axis, the vortex domains expand inside the segment following the structures formed at the interfaces and result in one vortex domain for short segments and two or more vortex structures for longer segments. When the field is applied perpendicular to the nanowire but along the anisotropy easy axis, transverse domains are formed (Figure 15e-(1), (2)). They are separated by vortex domain structures with the core at the surface pointing perpendicular to it (Figure 15e-(3)) [73]. This configuration can also be considered as a chain of multi-vortex domains with the cores pointing perpendicular to the nanowire axis and transverse domain walls in between [54].

**Figure 16:**
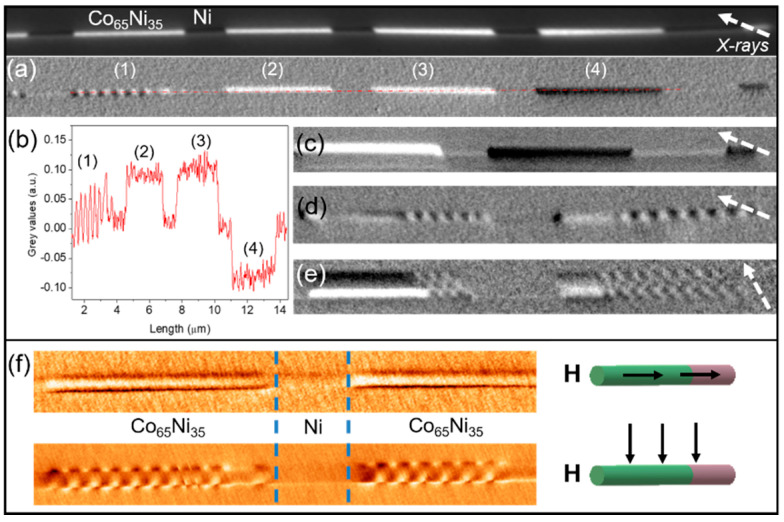
Chemical contrast of a multisegmented Co_65_Ni_35_/Ni nanowire (top panel). (**a**) XMCD-PEEM image of a Co_65_Ni_35_/Ni nanowire and (**b**) the contrast profile of CoNi segments in (**a**), labeled (1) to (4), with different magnetic structures. (**c**–**e**) XMCD-PEEM images of Co_65_Ni_35_/Ni nanowires showing different magnetic configurations. (**f**) MFM images taken in remanence (adapted from [75]), after applying a magnetic field of 1.8 T in the axial direction (top panel) and perpendicular to the nanowire (bottom panel).

In a similar CoNi/Ni system, the strength of magnetocrystalline anisotropy of CoNi segments was lowered by reducing the Co content in the alloy. Figure 16 presents Co_65_Ni_35_ 2.1 μm long segments separated by Ni segments with 800 nm in length. The diameter of the multisegmented structure is 130 nm. The Co_65_Ni_35_ segment composition, the quality of interfaces, and the crystallographic structure were determined by HRTEM, confirming that Ni segments crystallize in *fcc* structure along (110), while CoNi alloy seems to grow epitaxially onto Ni segments [75]. 

Figure 16 shows the chemical contrast (top panel) and magnetic images ((a)–(f)) of Co_65_Ni_35_/Ni nanowires taken in remanence state. Figure 16a presents the XMCD-PEEM image of a CoNi/Ni nanowire measured at Co L_3_-edge with the beam (marked by the dashed white arrow) at about 20 deg. with respect to the nanowire axis. The presented nanowire is formed by four main CoNi segments displaying two types of magnetic configurations visible at the surface of the nanowire. Due to the orientation of the beam to the nanowire axis, the XMCD is sensitive to the single longitudinal domain state as imaged in segments (2) to (4) and (c) with the magnetization oriented parallel (bright contrast) or antiparallel (dark contrast) to the X-rays (i.e., along the nanowire axis). In the other segments (1), we observed a periodic alternating contrast, similar to the transversal domains presented in Figure 14b and Figure 15c. However, in this orientation of the X-ray propagation vector, almost parallel to the nanowire axis, only the magnetic moments oriented in the direction of the beam (along the wire axis) are observed which excludes the possibility of imaging transversal magnetic domains in the CoNi segments. Figure 16b presents the contrast profile taken along the red dotted line in the nanowire presented in (a): periodic magnetic structures with 140 nm in length (left side) followed by longitudinal single domains with different orientations. Figure 16c,d show closed up XMCD-PEEM images of different parts of the multisegmented nanowire presenting either longitudinal single domain structure (c) or a mixture of vortex and periodical domains (d). The same type of mixed magnetic configuration is presented in Figure 16e, this time measured with the beam oriented almost perpendicular to the nanowire axis. This specific orientation of the X-rays, almost perpendicular to the wire, gives us access to the “shadow” which allows us a second set of information about the core of the measured wire. From the alternate bright/dark contrast on the wire (dark/bright on the shadow) we conclude that the magnetization in CoNi segments consists of a series of periodic transversal domains with about 90 nm in length, similar to those observed in Co_85_Ni_15_ segments. As revealed by micromagnetic simulations (Figure 15e) the magnetization between transversal domains consists of vortices with the core oriented perpendicular to the nanowire axis (the so-called “surface vortices”). This is in agreement with the XMCD data presented in Figure 16a-(1),d where the observed alternating dark/bright contrast at the surface of the wire is consistent with the surface vortices predicted by simulations (Figure 15e-(3)), black dotted squared area) and observed experimentally by Electron Holography in CoNi [54]. By varying the direction of the X-ray propagation vector with respect to the nanowire axis, XMCD-PEEM allows us to get information about both transversal magnetic domains (X-rays oriented perpendicular to the wire, Figure 16e) and the domain walls in between, surface vortices (X-rays oriented along the wire axis, Figure 16a,d).

The same magnetic configuration, i.e., single or periodic domains, was observed by MFM [75]. Here, the magnetic configuration of Co_65_Ni_35_ could be tuned by magnetic fields (saturating or demagnetizing) applied parallel or perpendicular to the nanowire axis [75]. Figure 16f presents the MFM pictures of the same multisegmented nanowire (two Ni segments and one CoNi segment), taken after the magnetic field was applied parallel (Figure 16f-upper panel) or perpendicular (Figure 16f-bottom panel) to the nanowire axis. Depending on the field history, the magnetization state of Co_65_Ni_35_ changes either in a single domain state (Figure 16f-upper panel) or periodic domains (Figure 16f-bottom panel).

The multisegmented wires with modulated anisotropy presented in this section show a complex interplay of different energetic contributions and geometry determining the resulting magnetic structures. This determines the different factors which should be taken into account for the design of magnetic nanowires with a certain magnetic configuration: geometry (diameter, length), the combined materials (interplay between shape and magnetocrystalline anisotropy), and field history. 

## 5. Discussion and Summary

Multiple cylindrical modulated magnetic nanowires with different materials and geometries were prepared by electroplating filling the pores of anodic aluminum oxide (AAO) membranes. The AAO membranes with modulated pores and various diameters were obtained by pulsed anodization in sulfuric or oxalic aqueous solution. The single element and alloy nanowires, as well as multisegmented ones, were grown inside the nanopores, at room temperature, by electrodeposition using different electrolytes.

The results show that in the case of nanowires with uniform diameter and small magnetocrystalline anisotropy the magnetic configuration at remanence consists of a single domain with open vortices at the ends. These nanowires (Ni and Fe-based) are interesting candidates to study the motion of domain walls.

Large magnetic anisotropy nanowires show a more complex behavior in terms of magnetic configurations. Co-based nanowires with an hcp crystallographic structure can be prepared with magnetocrystalline anisotropy almost perpendicular to the nanowire axis and typically present vortex domains with alternating chirality. The combination of an almost perpendicular to nanowire anisotropy and the circular symmetry promotes the spontaneous development of vortex domains with magnetic moments following a circumferential path at the surface but staying longitudinal in the core. Furthermore, the use of different alloys with tailored compositions can alter the magnetic configuration going from axial domains to a combination of vortex and transverse domains, as shown in the example of CoNi alloy nanowires with different compositions. The control between different structures in some cases can be achieved by perpendicularly applied magnetic fields.

Multisegmented/multilayered nanowires offer an additional route to control the magnetization pattern via confinement and pinning. Particularly, further engineering of the nanowires can be provided by creating modulations/constrictions in diameter with the aim to achieve the pinning of domain walls at the constrictions. By altering the geometry of the nanowire, imprinting geometrical modulations (notches/anti-notches, alternating segments with different diameters) along the nanostructure’s length the magnetic configuration changes. Although the inner magnetization stays unchanged (i.e., along the nanowire axis) surface chiral structures get pinned at the geometrical modulations distributed along the nanowire’s length. 

Both micromagnetic simulations and imaging revealed that in the case of modulated in geometry wires with high saturation magnetization, the magnetic configuration and pinning can be tailored further by playing with the diameter of the segments. Moreover, micromagnetic simulations show that when the diameter difference is large, we observe the formation of topologically protected structures, where the magnetization of the large-diameter segment forms a skyrmion tube with a core position in a helical modulation along the nanowire. This leads to an increase in the coercive field, as compared to the nanowires with uniform diameter, associated with the occurrence of a novel pinning type, i.e., “corkscrew” mechanism. 

The nanowires also can be prepared with a well-controlled multilayer structure with alternating magnetic or magnetic/non-magnetic materials. The former allows the modulation of the magnetic anisotropy, while the latter can be used for domain wall pinning or confinement of different domains.

Engineering the nanowires with alternating magnetic/non-magnetic segments provides a way to control magnetization confinement and interactions by tailoring the lengths of the ferromagnetic segment and the thickness of the non-magnetic layer. Indeed, the magnetization structure in nanowires of high saturation and short segments can change from the longitudinal domains in a single material nanowire to vortex configurations confined in the segments. Moreover, the magnetization can be easily controlled by magnetic fields as shown in FeCo segments. If the length of FeCo segments, separated by 30 nm Cu layers, is gradually increased a ratchet system is formed where the magnetization reversal in neighboring segments propagates sequentially in steps starting from the shorter segments, irrespectively of the applied field direction. For FeCo segments with a constant length, separated by 50 nm Cu layers, the segments present a single vortex state with alternating chirality.

On other hand, the magnetic anisotropy was modulated in cylindrical nanowires by alternating two ferromagnetic materials with different magnetocrystalline anisotropy. This strategy shows a rich and complex behavior. The XMCD-PEEM imaging of multisegmented Ni/CoNi nanowires reveals that the magnetic structure in the Ni segment is in a dominantly axial magnetic state while in CoNi segments it depends on their length. Although in an almost single domain configuration, Ni state appeared to have an important impact on the CoNi, through magnetic interaction and magnetochiral effects, as revealed by micromagnetic simulations. The field history is revealed as another important factor, responsible for the appearance of different domain patterns (vortex domains or perpendicular/transversal domains separated by vortices on the surface) in large CoNi segments. 

Future applications will require efficient manipulation of magnetic structures in individual nanowires. In this review, we presented many examples of how magnetic domain structure is changed by geometry, magnetocrystalline anisotropy, and applying field direction. At the same time, future energy-efficient applications will require the use of current, which is scarcely investigated. Here we presented the results of micromagnetic simulations in a Py nanowire under the simultaneous action of the magnetic field and spin-polarized currents. The results show that the current alone enlarges or reduces the open vortex structures formed at the ends of the nanowire depending on the rotational sense of the associated Oersted field. Large current densities can set the vorticity along the whole nanowire in the desired direction. The switching of magnetization in the core is solely achieved by the application of a simultaneous external field, lower than the coercive field of the nanowire. 

In conclusion, our results demonstrate a complex interplay of different energetic contributions and geometry determining the resulting magnetic configurations. We stress the importance of different factors which should be taken into account for the design of magnetic nanowires. Their tunability makes them excellent candidates for new applications where full control over the magnetization reversal is mandatory.

## Figures and Tables

**Table 1:** Materials and parameters used in electrodeposition.

Material	Electrolyte	Voltage
(1) Fe_30_Co_65_Cu_5_ [24]	0.05 M FeSO_4_·7H_2_O, 0.12 M CoSO_4_·7H_2_O, 0.16 M H_3_BO_3_, 0.01 M CuSO_4_·5H_2_O, 0.06 M C_6_H_8_O_6_	−1.8
(2) Fe_50_Co_50_ [24]	0.08 M CoSO_4_∙7H_2_O, 0.08 M FeSO_4_∙7H_2_O, 0.16 M H_3_BO_3_, 0.06 M C_6_H_8_O_6_	−1.8
(3) Ni	0.76 M NiSO_4_·6H_2_O, 0.17 M NiCl_2_·6H_2_O, 0.65 M H_3_BO_3_	−1.0
(4) Co_65_Ni_35_ [20]	0.09 M CoSO_4_·7H_2_O + 0.063 M CoCl_2_·6H_2_O +0.095 M NiSO_4_·7H_2_O + 0.084 M NiCl_2_·6H_2_O + 0.32 M H_3_BO_3_	−1.1
(5) Co_85_Ni_15_ [20]	0.12 M CoSO_4_·7H_2_O + 0.084 M CoCl_2_·6H_2_O + 0.064 M NiSO_4_·7H_2_O + 0.063 M NiCl_2_·6H_2_O + 0.32 M H_3_BO_3_	−1.2

**Table 2:** Materials parameters used in micromagnetic modeling: saturation magnetization, μ_o_M_s_, exchange stiffness, A_ex_, exchange-correlation length, l_ex_ = (2A_ex_/μoMs^2^)^1/2^, crystalline symmetry, first magnetocrystalline anisotropy constant, K_1_, and the direction of the magnetization easy axis with respect to the nanowire axis. Saturation magnetization and exchange stiffness for CoNi alloys have been obtained by linear interpolation with the Co content of the alloy [20].

Material	μ_o_M_s_ (T)	A_ex_ (pJ/m)	l_ex_ (nm)	Crystal Symmetry	K_1_ (kJ m^−3^)	Magnetization Easy Axis (e.a.)
Fe_20_Ni_80_ [34]	1.0	10.8	5.2	-	0	-
Co(111) [34]	1.76	13.0	3.3	Cubic	−75	parallel to nanowire axis
Co(100) [34]	1.76	13.0	3.3	Uniaxial	450	e.a. at 75°–88° with nanowire axis
Co-hcp [34,35]	1.76	30.0	4.9	Uniaxial	450	e.a. at 75°–88° with nanowire axis
Fe_30_Co_70_ [36]	2.0	10.7	2.6	Cubic	10	polycrystalline textured, e.a. at 45° vs. nanowire axis *
Ni(111) [34]	0.61	3.4	4.8	Cubic	−4.8	parallel to nanowire axis
Co_85_Ni_15_ [20,35,37,38]	1.60	26.0	5.1	Uniaxial **	350	e.a. at 65–88° with nanowire axis
Co_65_Ni_35_ [20,35,37,38]	1.35	15.0	4.5	Uniaxial **	260	e.a. at 65° with nanowire axis
Co_35_Ni_65_ [20,35,37,38]	1.01	10.0	5.0	Cubic	2	parallel to nanowire axis

* In each grain, one easy axis (e.a.) is randomly set on the surface of a cone of 45 degrees with respect to the nanowire axis, and the second e.a. is placed with random orientation in the plane, normal to the first axis [39]. ** Polycrystalline structure was also used with no significant difference with the uniaxial anisotropy.
